# Immunotherapy in Advanced Prostate Cancer—Light at the End of the Tunnel?

**DOI:** 10.3390/ijms23052569

**Published:** 2022-02-25

**Authors:** Gunhild von Amsberg, Winfried Alsdorf, Panagiotis Karagiannis, Anja Coym, Moritz Kaune, Stefan Werner, Markus Graefen, Carsten Bokemeyer, Lina Merkens, Sergey A. Dyshlovoy

**Affiliations:** 1 Department of Hematology and Oncology, University Medical Center Hamburg-Eppendorf, Martinistrasse 52, 20246 Hamburg, Germany; w.alsdorf@uke.de (W.A.); p.karagiannis@uke.de (P.K.); a.coym@uke.de (A.C.); m.kaune@uke.de (M.K.); c.bokemeyer@uke.de (C.B.); s.dyshlovoy@uke.de (S.A.D.); 2 Martini-Klinik, Prostate Cancer Center, University Medical Center Hamburg-Eppendorf, Martinistrasse 52, 20246 Hamburg, Germany; graefen@uke.de; 3 Department of Tumor Biology, University Medical Center Hamburg-Eppendorf, Martinistrasse 52, 20246 Hamburg, Germany; st.werner@uke.de (S.W.); l.merkens@uke.de (L.M.); 4 Laboratory of Pharmacology, A.V. Zhirmunsky National Scientific Center of Marine Biology, Palchevskogo Str. 17, 690041 Vladivostok, Russia

**Keywords:** immunotherapy, advanced prostate cancer, PD-L1, CTL-A4, immune checkpoint inhibitors, BiTE, CAR-T cells, vaccination, tumor microenvironment

## Abstract

Immunotherapeutic treatment approaches are now an integral part of the treatment of many solid tumors. However, attempts to integrate immunotherapy into the treatment of prostate cancer have been disappointing so far. This is due to a highly immunosuppressive, “cold” tumor microenvironment, which is characterized, for example, by the absence of cytotoxic T cells, an increased number of myeloid-derived suppressor cells or regulatory T cells, a decreased number of tumor antigens, or a defect in antigen presentation. The consequence is a reduced efficacy of many established immunotherapeutic treatments such as checkpoint inhibitors. However, a growing understanding of the underlying mechanisms of tumor–immune system interactions raises hopes that immunotherapeutic strategies can be optimized in the future. The aim of this review is to provide an overview of the current status and future directions of immunotherapy development in prostate cancer. Background information on immune response and tumor microenvironment will help to better understand current therapeutic strategies under preclinical and clinical development.

## 1. Background

Prostate cancer (PCa) is the most common malignancy and second leading cause of cancer-related death amongst men in the Western world [1]. 

In the metastatic setting, tremendous progress has been made in recent years. Thus, in metastatic hormone-naïve PCa (mHNPC), combinational treatment with androgen-deprivation therapy (ADT) and new hormonal agents (NHA) or chemotherapy with docetaxel is recommended [2]. Interesting new data on triple therapy consisting of ADT, docetaxel, and abiraterone or darolutamide compared with hormone chemotherapy alone showed a clinically significant survival benefit and will set a new standard, especially for patients with high-risk constellations [3,4]. In the castration-resistant setting, additional treatment options include cabazitaxel, PARP inhibitors, and PSMA radioligand therapy [5,6,7,8]. However, prognosis of advanced-stage patients is still poor due to the development of resistance to currently available standard therapies. Therefore, new therapeutic approaches are urgently needed.

The idea to utilize the patients’ immune system to fight tumors has revolutionized the field of anticancer therapy within the last decade [9]. In fact, immunotherapeutic approaches have been triumphant in several highly immunogenic cancers, often called “hot tumors” (such as melanoma, renal carcinoma, and lung cancer, among others) [10,11,12]. Thus, immune-checkpoint monotherapies or combination regimens targeting cytotoxic T lymphocyte antigen 4 (CTLA-4) and/or the programmed death-1 (PD-1)/PD-1 ligand (PD-L1) axis have become an integral part of various first-line standard therapies in a variety of malignancies. In contrast, so-called “cold tumors”, such as prostate cancer (PCa), possess an immunosuppressive tumor microenvironment (TME) resulting in a very restricted response to immunotherapy. In fact, immunotherapy has so far been generally disappointing in PCa. To date, despite intensive efforts, sipuleucel T is the only immunotherapeutic agent that has achieved a significant survival benefit in a randomized Phase 3 clinical trial (see Section 3.1.1). [13]. Currently investigations are conducted to make the immunologically cold PCa accessible to immunotherapy by manipulating the tumor microenvironment as well as implementing new immunological treatment strategies with bispecific T cell engagers (BiTEs) or CAR-T cells.

In this review, we discuss mechanisms contributing to immune response and resistance of PCa, summarize the different treatment approaches and study results available, and provide an overview of the current study landscape.

## 2. Immune Response and the Role of the Microenvironment in Prostate Cancer

### 2.1. Intrinsic Factors Influencing Immune Response

#### 2.1.1. Tumor Mutational Burden and Neoantigen Expression

**Figure 1:**
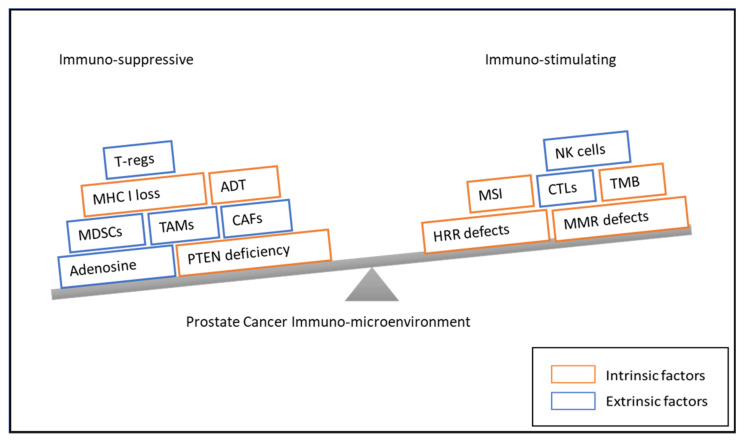
Immunosuppressive and -stimulating factors influencing immune response in advanced prostate cancer. Abbreviations: T-reg: regulatory T cell, MHC I: major histocompatibility complex I, ADT: androgen-deprivation therapy, MDSC: myeloid-derived suppressor cell, TAM: tumor-associated macrophage, CAF: cancer-associated fibroblast, PTEN: phosphatase and tensin homolog, NK cell: Natural Killer cell, MSI: microsatellite instability, CTL: cytotoxic T lymphocyte, TMB: tumor mutational burden, HRR: homologous recombination repair, and MMR: mismatch repair.

The recognition of neoantigens is a central mechanism mediating antitumor immunity (Figure 1). Neoantigens result from non-synonymous mutations translating into aberrant proteins. These are presented to the immune system, which consequently recognizes the tumor cell as “foreign”. Multiple studies have confirmed that response to immunotherapy is associated with tumor mutational burden (TMB) in a variety of tumor entities [14]. TMB in PCa is low, especially in comparison to immunological “hot” tumors such as melanoma or non-small cell lung cancer [15]. The resulting low number of neoantigens is considered one of the tumor intrinsic factors contributing to the low response rates to immunotherapy in PCa [15]. Although TMB has been shown to be a good predictor to immunotherapy response, there are still patients with antitumor responses despite a divergent TMB result (Figure 1). In addition, PCa is characterized by a comparatively high amount of structural variants, such as indels and insertions or fusions, which also lead to expression of neoantigens [16]. A recent study demonstrated a correlation of high-fusion burden in PCa with increased immune infiltration, PD-L1 expression on immune cells, and immune signatures, representing activation of T cells and M1 macrophages [17].

#### 2.1.2. Expression of Programmed Death Ligand-1 (PD-L1)

Generally, PD-L1 expression levels in PCa are lower compared with other cancers, although up to one-third of mCRPC tumors may show some PD-L1 expression on tumor cells [18]. The range of reported PD-L1 expression in PCa is wide, varying from no expression to over 90% in some patients. For example, immunohistochemical expression of PD-L1 has been detected in 29% acinar PCas, 7% ductal PCas, and 46% neuroendocrine PCas [19]. PD-L1 expression on TILs was found in 9%–14.6% of the cases [18,20,21,22,23,24,25]. While some studies observed a correlation between Gleason Score and PD-L1 expression [21,23], this was not confirmed by others [18,22,24]. Data on the correlation of PD-L1 expression with gene alterations related to tumor progression and aggressiveness are not yet comprehensively available (reviewed by Palicelli et al. [26]). Indeed, in patients with MSI-H/dMMR-positive disease, PD-L1 expression was detected in only about 12%. Patients with a *PTEN* deletion carry increased PD-L1 expression in about 10%, whereas some enrichment is apparently found in *SPOP*-mutated PCa. Data on expression in homologous recombination repair (HRR) defects remain to be comprehensively collected [26]. Although study results have been inconsistent, increased PD-L1 expression has been associated with a higher risk of biochemical recurrence or metastatic progression [21,24,27,28]. This substantial variability can also be found in PD-L1 as a predictor of immunotherapeutic responses (Figure 1).

#### 2.1.3. DNA Repair Defects

Loss of function of DNA damage repair (DDR) genes potentiates TMB and genomic instability. The mismatch repair (MMR) system repairs single base substitutions and short indels, for example during DNA synthesis. Defects in the MMR system can lead to point mutations, frameshifts, and the phenomenon of microsatellite instability (MSI). A recent study found 3% of MSI high tumors in an unselected cohort of PCa patients. This percentage can rise up to 12% in advanced PCa [29,30]. Indeed, MSI seems to be acquired in late disease stages, while about 22% of affected patients already harbor germline mutations [29]. MMR-deficient PCa shows higher immune infiltration and an increased response to immunotherapy compared to MMR-proficient tumors [29,31,32,33] (Figure 1). Still, about half of the patients with high MSI do not respond to immune therapy. However, causes of this primary resistance remain to be examined [29].

Mutations in other DDR genes occur more frequently. These include alterations in the HRR pathway genes, e.g., *BRCA2* and *ATM*, among others [34]. Analysis of patient tissue revealed that *BRCA2*-mutated tumors had more T cells within the tumor compared to the extratumoral tissue. However, these tumors were simultaneously infiltrated with more regulatory T cells (Treg) compared to *BRCA2* wild-type tumors [35]. Mechanistically, impaired DNA repair due to *BRCA2* loss leads to cytosolic DNA fragments. In turn, these activate a cGAS/STING-mediated interferon response [36]. However, the inconsistency surrounding clinical outcomes in patients bearing cancers with DDR alterations and treated with immune checkpoint inhibitors questions its relevance as a biomarker [37,38].

#### 2.1.4. Inactivation of PTEN

The loss of tumor-suppressor protein PTEN, especially in combination with inactivation of RB1 and/or TP53, has been recognized as one of the signs of aggressive variant PCa (AVPC) [39,40]. PTEN inactivation is present in approximately 20% of primary tumors and in around 40% of advanced PCa [41]. In immune response, functional PTEN has been associated with activation of pro-inflammatory INF1 and NF-kB pathways [42,43]. In line with this, *PTEN*-deficient PCa tumors have higher Treg cell infiltration, which is associated with an immunosuppressive TME of these tumors as well as resistance to immunotherapy [44] (Figure 1). Recently, increased recruitment of myeloid-derived suppressor cells (MDSCs) has been described in *PTEN*-deficient tumors [45,46]. Based on these findings, targeting of MDSCs in PCa tumors expressing mutant or null *PTEN* is currently under clinical investigations. 

#### 2.1.5. Androgen Receptor Signaling

Androgen receptor (AR) signaling is critical for progression and survival of normal and malignant prostate cells [47]. ADT decreases the levels of circulating testosterone (via e.g., inhibition of the GnRH receptor) and is a central component of systemic therapy for advanced PCa. Direct inhibition of the AR or of androgen synthesis are additional therapeutic approaches to successfully influence AR signal transduction [48].

The effect of ADT on tumor immunogenicity is complex and, in some cases, controversial. In AR-dependent tumors, an androgen withdrawal initially results in an increase of TILs and reduction of Treg cells [49,50,51]. In vivo studies have indicated direct immunomodulatory effects of ADT on PCa TME, though the results were strongly dependent on the utilized models [49,50]. Moreover, development of castration resistance under continuous ADT treatment seems to correlate with simultaneous increase of immune tolerance [52]. Additionally, ADT can impair adaptive immune response by inhibition of T cell function, which may be related to the off-target effects of AR antagonists on the γ-aminobutyric acid receptor [53]. Thus, in patients suffering from localized PCa, treatment with ADT in combination with GVAX (cell-based vaccine) resulted in promoted infiltration of CD8 ^+^ T cells accompanied by a simultaneous increase of the immunosuppressive Treg cell population [54]. In addition, the upregulation of immune checkpoints and TILs has been observed in samples obtained from patients receiving ADT therapy [55]. 

### 2.2. The Role of Tumor Microenvironment in Prostate Cancer

The TME of PCa is highly immunosuppressive due to infiltration of regulatory T cells, tumor-associated macrophages (TAM), and MDSCs, the cytokine milieu secreted by tumor stromal cells and fibroblasts, as well as the production of adenosine via prostatic acid phosphatase [56,57]. 

#### 2.2.1. The Tumor Cytokine Milieu

Cytokines regulate and facilitate immune response to different stimuli, including tumor development [58]. In PCa progression as well as during PCa, directed immunotherapy cytokines play a dual role [59]. On the one hand, in local chronic inflammatory conditions the production of pro-inflammatory cytokines leads to the promotion of CTL infiltration into the tumor; on the other hand, the traffic of immunosuppressive MDSC and Treg cells is increased due to the stimulation by IL-1β and IL-2 cytokines (reviewed in [59]). Moreover, tumor necrosis factor-α (TNF-α) and IL-17 were found to induce expression of immunosuppressive PD-L1 on PCa cell surface contributing to immune resistance [60]. Accordingly, increased expression of anti-inflammatory cytokines in PCa was accompanied by substantial suppression of CTL infiltration and activity [61]. Thus, the levels of anti-inflammatory cytokines IL-4, IL-6, and IL-10 were elevated in serum of patients suffering from hormone-refractory PCa [62] and associated with increased PSA levels. Additionally, it has been shown in patients that secretion of immunosuppressive cytokines such as TGF-β and IL-10 facilitate a weak antitumor immune response and condition a poor treatment prognosis [61,63,64].

#### 2.2.2. Myeloid-Derived Suppressor Cells (MDSCs)

The release of inflammatory cytokines leads to accumulation (recruitment) of MDSC in TME as well as differentiation of myeloid cells to MDSC [65]. MDSC are a population of immature myeloid cells that exhibit immune-suppressive effects on T cells and NK cells and therefore are considered to be an important mechanism of immunotherapeutic resistance in PCa (reviewed in [66,67]) (Figure 1). MDSCs promote recruitment of Tregs that suppress the function of CTLs, and can differentiate to monocytes or neutrophils and then further to M2 macrophages, which also inhibits CTL activity [68]. Consequently, low T cell recruitment and reduced activity result in inefficacy of immunotherapeutic strategies in PCa. Additionally, MDSC promote growth and survival of PCa cells via other mechanisms unrelated to immunosuppression [45,69]. Increased MDSC infiltration in tumor tissue and blood has been reported in patients with an accumulation from localized to metastatic PCa [70]. This correlated positively with Treg levels and negative prognostic markers [71]. Moreover, a large population of MDSCs has been found in the metastatic regions of the bones, which are the main metastatic sites of PCa [72,73]. Remarkably, the elimination of MDSC significantly improved T cells infiltration and promoted anticancer effects of immune checkpoint inhibitors (CI) in the pre-clinical setting [74]. In line with this, S100A9 inhibitor tasquinimod, a MDSC-targeting drug, exhibited a clear benefit for progression-free survival (PFS), even though it failed to improve overall survival (OS) in mCRPC [75].

#### 2.2.3. Tumor-Associated Macrophages (TAMs)

TAMs are a key component of the inflammatory TME. These highly heterogeneous cells originate either from resident tissue-specific macrophages or newly recruited monocytes [76]. Generally, they can be classified in a tumor-inhibiting M1 phenotype or a tumor-promoting M2 phenotype. However, mixed phenotypes as well as a phenotype switching upon stimulation including the development of subpopulations with differences in antigen expression have been observed [77]. TAMs can stimulate tumor cell proliferation, migration and genetic instability [78]. In PCa, an increased TAM density has been associated with higher Gleason score and shorter cancer-specific survival [77,79,80]. Of note, increased activation of osteoclast related pathways has been associated with TAMs as well [81]. This is of great interest because PCa metastasizes mainly to the bones [82]. A targeted approach of TAMs seems to be a reasonable therapeutic procedure and is currently being investigated mainly in pre-clinical models [83].

#### 2.2.4. Stromal Cells

TME consists of various populations of stromal cells. Apart from innate and adaptive immune cells, it includes non-immune cell like fibroblasts, endothelial cells, adipocytes, mesenchymal stromal cells (MSCs), and pericytes [84]. In terms of tumorigenesis, the cancer-associated fibroblasts (CAFs) and endothelial cells have been assumed to be most relevant, with the latter being involved in promotion of neo-angiogenesis, and thus local progression and hematogenous dissemination [85]. CAFs are the most abundant stromal cells represented in the TME [86]. These cells may be of a different origin and are known to play an important role in tumorigenesis via promotion of angiogenesis, remodeling of the extracellular matrix (which supports tumor cells invasion), and expression of growth factors as well as cyto- and chemokine, thereby mediating a cross-talk with PCa cells [86,87]. The most important factors involved in this interaction are bFGF, PDGF, and TNF-α, as well as MMPs and VEGF. These mediators promote acquisition of stem cell properties as well as an epithelial–mesenchymal transition (EMT) of the PCa cells, ultimately leading to a more aggressive phenotype and increased drug resistance [88]. Moreover, the proliferation of CAFs leads to an alteration of vascular structure of the tumor and the surrounding tissues, which in turn results in development of hypoxia and local inflammation, and consequently leads to the infiltration of immunosuppressive MDSCs and Treg cells in TME [89].

Additionally, CAFs can directly inhibit the antitumor functions of cytotoxic lymphocytes. Thus, CAFs abrogate the cytotoxic function of NK cells due to the expression of PGE2 and IDO by CAFs, which leads to the inhibition of cytotoxic function and inactivation of NK cells [90,91]. Additionally, CAFs express MMPs, which degrade MICA/B on the surface cancer cell (the NKG2D ligands) and therefore abrogate a NKG2-dependent cytotoxicity of NK cells [90]. CAFs can also inhibit the function of cytotoxic T cells via various mechanisms, such as the following: (a) IL-1-mediated expression of PD-L1; (b) overexpression of CD39 and CD73, which results in generation of high amounts of immunosuppressive adenine in the TME; (c) release of lactate by the glycolytic CAFs, which affects polarization and function of cytotoxic T lymphocytes; and (d) activation of TGF-β signaling, which is correlated with the exhaustion of T cells and was associated with poor response to anti-PD-1 therapy [87] (Figure 1).

#### 2.2.5. Adenosine in PCa

Adenosine is a small molecule, which, among others, acts as an anti-inflammatory mediator. This effect is executed mainly via adenosine-dependent activation of A2_a_/A2_b_ receptors expressed on the surface of T cells resulting in their inactivation. In normal settings, this mechanism helps to protect healthy tissues from the damage induced by the inflammatory processes [92]; however, within the tumor microenvironment, the immunosuppressive effects of adenosine results in tumor immune evasion [93]. Thus, adenosine suppresses the cytotoxic function of TILs [93,94]; additionally, it can increase Treg cells and MDSCs, increase the number of tumor-promoting and angiogenic fibroblasts, and inhibit functionality of dendritic cells [95,96,97,98]. 

In physiological conditions, adenosine is produced from ATP via its consecutive dephosphorylation catalyzed by ectonucleotidases like CD39, CD73, prostatic acid phosphatase (PAP), and alkaline phosphatase [99]. Interestingly, extracellular ATP functions such as the danger-associated molecular pattern (DAMP) promotes immune responses, while in its dephosphorylated form adenosine exhibits immunosuppressive properties, therefore stabilizing and balancing an immune response [98]. Of note, PAP is highly expressed in PCa tissues [100]. Therefore, PAP targeting may represent an attractive mechanism to increase the efficacy of immunotherapy by the inhibition of adenosine production. However, vaccination trials targeting PAP have been rather disappointing (see below). Early clinical trials are now investigating efficacy of the A2_a_ and/or A2_b_ receptor antagonists and CD73 ectonucleotidase inhibitors in PCa [101,102].

## 3. Immunotherapeutic Treatment Approaches

### 3.1. Vaccine-Based Treatment Modalities

**Figure 2:**
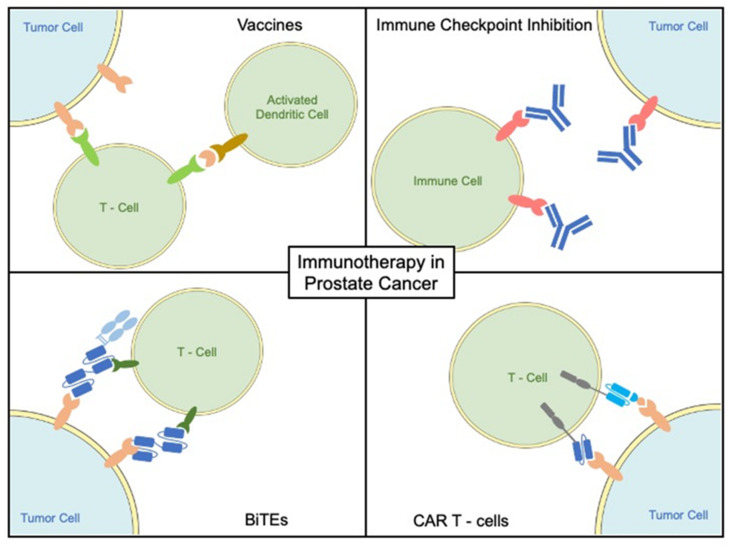
Immunotherapeutic approaches in advanced prostate cancer.

Vaccination strategies are based on activation of the immune system against specific tumor-associated antigens (TAA), e.g., prostate-specific antigen (PSA), prostate-specific membrane antigen (PSMA), prostatic acid phosphatase (PAP), or prostate stem cell antigen (PSCA) [103,104]. Applied with co-stimulatory molecules or loaded on patients’ immune cells, vaccines are recognized by APC cells, processed, and presented with MHC class 1 molecules to CD8 ^+^ cells, turning them into cytotoxic T effector cells (Figure 2) [105]. Various vaccination approaches have been tested in PCa to date including cell-based vaccines, peptide vaccines, viral/bacterial vaccines, as well as DNA- or RNA-based vaccines.

#### 3.1.1. Cell-Based Vaccines

Cell-based vaccines are derived from patients’ own tumor cells (autologous) or from tumor cell lines (allogeneic). For example, the granulocyte-macrophage colony-stimulating factor-transduced allogeneic prostate cancer cell vaccine (GVAX) was synthesized from two prostate cancer cell lines, LNCaP and PC-3, and transfected with a human GM-CSF gene. However, GVAX failed to succeed in two phase 3 clinical trials applied as monotherapy or in combination with docetaxel (VITAL-1; VITAL-2) [106]. In contrast, sipuleucel-T, a dendritic cell (DC)-based immunotherapy, improved overall survival (OS) in PCa and was approved by the FDA in 2010 for the treatment of asymptomatic or minimally symptomatic mCRPC. For the synthesis of sipuleucel-T, patient-derived peripheral blood mononuclear cells (PBMCs) are stimulated ex vivo with a recombinant fusion protein consisting of prostatic acid phosphatase and the granulocyte-macrophage colony-stimulating factor. Afterwards, the PBMCs are reinfused every 2 weeks for a total of three infusions. Results from the phase 3 study IMPACT showed a relative reduction in the risk of death of 22% (HR 0.78; 95% CI 0.61 to 0.98; *p* = 0.03) and a significant improvement of 4.1 months in median OS in the sipuleucel-T group compared to the placebo group. Immune responses to the immunizing antigen were detected after sipuleucel-T treatment [13]. However, in Europe, the marketing authorization for sipuleucel-T was withdrawn at the request of the marketing authorization holder, limiting its clinical application. Currently, different combinational approaches are under investigation, including promising results reported on a combination with radium-223 (NCT02463799) [107]. Despite this initial success, further DC vaccines have failed to succeed [108]. For example, in the phase 3 VIABLE trial, the addition of autologous DC-based immunotherapy to docetaxel and prednisone did not prolong overall survival compared to chemotherapy alone in mCRPC patients [109]. In addition, no differences in the secondary efficacy end points (rPFS, time to PSA progression, or skeletal-related events) were reported.

#### 3.1.2. Peptide Vaccines

Peptide vaccines are produced by using specific epitope subunits of TAA. Comparable to cell-based vaccinations, peptide vaccination strategies can be based on common TAA or individual peptide structures. To date, different peptide vaccines have been evaluated in early phase 1/2 clinical trials, with some thoroughly promising initial results. For example, targeting Ras homolog gene family member C (RhoC) induced a potent and long-lasting T cell immunity in the majority of patients who had previously undergone radical prostatectomy (NCT03199872) [110]. Additionally, human telomerase reverse transcriptase (hTERT) peptide vaccine UV1 in combination with GM-CSF induced specific immune responses in the majority of mHNPC patients unselected for HLA type with tolerable adverse events [111]. Additional early phase clinical trials are currently recruiting, e.g., with B-cell lymphoma extra-large protein (Bcl-xl) 42-CAF09b (Bcl-xl_42: peptide fragment; CAF09b: adjuvance to enhance immunostimualtion) (NCT03412786; Table 1). 

Personalized peptide vaccination (PPV) uses multiple cancer peptides based on the pre-existing host immunity. In a randomized phase 2 trial, HLA-type specific peptides were chosen for vaccination based on the evaluation of both antipeptide IgG levels in plasma and CTL precursors in PBMCs of each patient (a maximum of four reactive proteins) and compared to low dose dexamethasone. Remarkably, PSA-PFS and median OS were significantly longer in the vaccination group compared to the dexamethasone group (PSA-PFS: 22.0 vs. 7.0 months; *p* = 0.0076; OS: 73.9 vs. 34.9 months; *p* = 0.00084), respectively [112]. Currently, a phase 1 study is evaluating PPV in combination with immune modulator Poly-ICLC, and hematopoietic cytokine CDX-301 in the adjuvant setting (Table 1; NCT05010200).

#### 3.1.3. Viral/Bacterial-Based Vaccines

PROSTVAC (PSA-TRICOM) is a viral vector-based vaccine using two different poxviral vectors for human PSA (PROSTVAC-V and -F). Additionally, these vectors include three co-stimulatory molecules for T cells (TRICOM) to enhance immune response. However, promising results of a phase 2 study [113] did not transfer into the phase 3 setting (NCT01322490), which compared patients receiving PROSTVAC (*n* = 432) or PROSTVAC plus granulocyte-macrophage colony-stimulating factor (*n* = 432) to placebo (*n* = 433). Neither treatment regimen had an impact on OS or on the number of patients alive without events [114]. Accordingly, FDA or EMA approval was not granted; however, combinational therapies are currently under investigation. Thus, PROSTVAC combined with CIs ipilimumab/nivolumab as well as a the neoantigen DNA vaccine are currently evaluated in mHNPC in a phase 1 clinical trial (NCT03532217). In addition, the efficacy of PROSTVAC co-applicated with or followed by docetaxel is determined in first-line treatment of mHSPC patients in a phase 2 clinical trial (NCT02649855). 

In addition to viruses, bacterial microorganisms can also serve as a source for vaccines. Therapeutic approaches using live tumor-targeting bacteria can either be applied as a monotherapy or complement other anticancer therapies to achieve better clinical outcomes [115]. In general, a major concern in the field of bacterial-based cancer therapies (BBCT) is toxicity due to associated toxins, which may cause side effects, similar to infections [116].

In the phase 1/2 trial KEYNOTE-046, DXS-PSA, an attenuated Listeria monocytogenes-based immunotherapy targeting PSA is examined in combination with pembrolizumab in mCRPC. Overall, 43% of the patients achieved a PSA reduction with a median OS of 33.6 months (NCT02325557) according to Stein et al. [117]. 

However, while virus-based immunotherapy is on the rise due to its relatively rapid and relatively uncomplicated production, the acceptance and implementation of BBCT is not yet at this scale. In addition to toxicity, cultural stigmas must be addressed before any decisive progress will be made. Recruiting trials on viral- and bacterial-based vaccines are shown in Table 1.

#### 3.1.4. DNA and RNA Vaccines

DNA vaccines serve as vehicles for in vivo transfection and antigen production. They consist of a plasmid DNA that encodes the antigen of interest under the control of a eukaryotic promoter [118]. To date, most DNA vaccines are focusing on antigens specific for PCa (e.g., PAP, PSA, or AR), while targeting of other tumor-specific mutation-associated neoantigens is challenging due to the rather low TMB of PCa. A cancer vaccine containing plasmid DNA encoding human PAP (pTVG-HP) has been investigated in several phase 1/2 trials. In these trials, multiple vaccinations were required to maintain an immune response, and still most patients did not benefit [118]. Thus, in a phase 2 clinical trial on patients with non mHNPC and biochemical recurrence, only a subgroup (having a rapid PSA doubling time) was identified to have an improved outcome concerning 2-year metastases-free survival (NCT01341652) [119]. Studies using PSA or PSMA as TAAs have been similarly disappointing so far. However, a combination of INO-5150 (a synthetic DNA therapy with plasmids encoding for PSA and PSMA) and INO-9012 (a synthetic DNA plasmid encoding for IL-12) showed first promising results in a phase 1/2 trial. In fact, a correlation of CD38 ^+^ and perforin ^+^ co-positive CD8 ^+^ T cell frequency to attenuated PSA rise for patients with biochemical recurrent non mHNPC was found [120]. In addition, the DNA vaccine pTVG-AR encoding the AR ligand binding has been evaluated in a phase 1 study (NCT024117869). Patients who developed T cell immunity had a significantly prolonged PSA-PFS compared with patients without immunity (HR = 0.01; 95% CI, 0.0–0.21; *p* = 0.003) [121]. Based on these findings, further investigations in combination with CIs have been initiated (NCT04989946; NCT04090528; Table 1).

Rapid development potentials and cost-effective manufacturing are the advantages of mRNA vaccines [105]. However, despite promising first signals in phase 1 clinical trials, follow-up investigations have been rather disappointing. Thus, standard therapy in combination with CV9104, a sequence-optimized, free, and protamine-complexed mRNA vaccine encoding the antigens PSA, PSMA, PSCA, STEAP1, PAP, and MUC1, failed to improve OS in mildly or asymptomatic mCRPC compared to standard therapy alone [122]. Another mRNA vaccine, W_pro1, which targets five antigens expressed in de novo and metastatic PCa and stably complexed with liposomes, is currently under clinical investigation in combination with PD-1 inhibitor Cemiplimab (NCT04382898, Table 1).

### 3.2. Checkpoint Inhibitors

Although the initial trials of immune CI in unselected PCa patients [123,124] failed to demonstrate significant clinical benefit (Figure 2), individual PCa patients showed impressive and durable responses. This raises the hope that immunotherapy could be a potential treatment option after an appropriate biomarker-based preselection [125,126].

#### Checkpoint Inhibitor Monotherapy

##### Anti-CTLA-4 Antibodies

Treatment with the anti-CTLA-4 antibody ipilimumab failed to demonstrate an OS benefit in two phase 3 clinical trials in patients with mCRPC (CA184 043 and CA184 095). Initially, ipilimumab was evaluated in docetaxel-pretreated mCRPC patients who had at least one bone metastasis. All patients received bone-directed radiotherapy (8 Gy in one fraction) followed by four courses of ipilimumab (10mg/kg) or placebo every three weeks. The primary endpoint was not reached with a median OS of 11.2 months (95% CI 9.5–12.7) with ipilimumab versus 10.0 months (8.3–11.0) with placebo (hazard ratio (HR) 0.85, 0.72–1.00; *p* = 0.053). However, in long-term analyses, a piecewise hazard model showed an improvement over time with a HR of 1.46 for the first five months, but onlyn 0.6 beyond 12 months with two to three times higher survival rates at 3 years and beyond for ipilimumab [127]. Next, ipilimumab monotherapy was evaluated in mild or asymptomatic mCRPC patients without visceral metastases prior to chemotherapy. Using the same regimen as in the previous trial, there was again no survival benefit for immunotherapy with a median OS of 28.7 months (95% CI, 24.5 to 32.5 months) in the ipilimumab arm compared to 29.7 months (95% CI, 26.1 to 34.2 months) in the placebo arm (HR 1.11; 95.87% CI, 0.88 to 1.39; *p* = 0.3667). Nevertheless, improved PFS and PSA response rates suggested antitumor activity in a patient subset [123].

Interestingly, exceptional clinical benefit has been reported in some patients with long-term responses, raising the question for appropriate selection criteria [125]. Recently, a single-center phase 2 clinical trial was conducted to address the impact of T cell responses to cancer neoantigens for an effective antitumor response to ipilimumab in mCRPC patients. Therefore, patients were assigned to two predominant categories depending on radiographic and/or clinical PFS (rcPFS). The favorable group had a rcPFS > 6 months and an OS > 12 months (*n* = 9), while the unfavorable cohort was characterized by a rcPFS < 6 months and an OS < 12 months (*n* = 10). Remarkably, in the pretreatment tumors, the IFN-γ response pathway signature was higher in patients in the favorable cohort compared to the unfavorable cohort. In addition, the favorable cohort had higher T cell gene signatures, including those of cytotoxic and memory T cells. Of note, an increased TMB was not associated with improved clinical responses to ipilimumab in this small number of patients. T cell responses to PSMA, PAP, and cancer neoantigens were only observed in patients within the favorable cohort [128].

##### PD-1/PD-L1 Inhibitors

Targeting the PD-1/PD-L1 axis alone has resulted in limited success in an unselected patient population [129]. Thus, in a phase 1/2 basket trial, nivolumab showed a significantly lower response in patients with PCa (*n* = 17) compared with those suffering from non-small cell lung cancer, melanoma, or renal-cell carcinoma (NCT00730639) [130]. 

KEYNOTE-028, a phase 1b multicenter basket trial with PD-1 inhibitor pembrolizumab included patients with advanced PCa who had progressed on standard therapy and had measurable disease per RECIST v1.1 as well as a positive PD-L1 expression in ≥1% of tumor or stromal cells (*n* = 23). In this small cohort of patients, a remarkable ORR of 17.4% and SD of 34.8% were reported, with an average response duration of 13 months (NCT02054806) [131]. Next, pembrolizumab was evaluated in KEYNOTE-199, a five-cohort, open-label, phase 2 study. In cohorts 1–3, patients with mCRPC treated with docetaxel and one or more of the targeted endocrine therapies were enrolled (cohort 1 and 2: RECIST measurable PD-L1–positive and PD-L1–negative disease; cohort 3: bone-predominant disease, regardless of PD-L1 expression). ORR and DCR were low with 5% and 10% in cohort 1 and 3% and 9% in cohort 2, respectively. In patients with predominant bone disease a DCR of 22% was reported. Of note, comparable to other entities, responses that did occur were durable. Among the nine patients with RECIST-measurable disease who achieved complete or partial response, five had responses ongoing at data cutoff with a median duration of 16.8 months (NCT02787005) [130]. 

The PD-L1 antibody atezolizumab was evaluated in 35 mCRPC progressive patients on sipuleucel-T or enzalutamide in a phase 1 trial. Two patients reached a PR, with one of those patients inheriting a MSH2 and MSH6 deletion, therefore being considered as MMR deficient. A 50% PSA response was reached by 8.6%, with a general median OS of 14.7 months and a 1-year OS rate of 52.3%. Although biomarker analyses showed that atezolizumab activated immune responses, no consistent biomarker linked to treatment efficacy was found [132]. 

Improved efficacy of PD-1/PD-L1 inhibition has been reported in PCa patients harboring MSI high tumors, with radiographic responses in 36% and 50% PSA decline in 54% of the cases, with most patients reaching long-term disease control (6).

In contrast, data on the impact of HRD on immune response are less clear. In KEYNOTE-199, a conditionally assessable tendency of higher pembrolizumab efficacy with a 11% ORR has been reported in patients with BRCA1/2 or ATM alterations compared to 3% in men without HRD defects (3). Currently, ImmunoProst is evaluating nivolumab in HRD-positive mCRPC (NCT03040791; Table 2).

Cycline-dependent kinase 12 (CDK12) and its loss of function due to biallelic mutation (prevalent in up to 6.9% of mCRPC patients) results in severe genomic instability, followed by a high load of immunogenic neoantigens because of widespread gene fusions. In a retrospective analysis, a PSA response was reported in 33% heavily pre-treated patients treated with a PD-1 inhibitor [133]. This immunogenicity might be a potent predictive biomarker for further investigation of immunotherapeutic efficacy in mCRPC (11). The IMPACT trial is currently evaluating a combinational CTL-A4/PD-1 inhibition in metastatic cancer harboring a loss of CDK12 function (NCT03570619; Table 2).

### 3.3. Checkpoint Inhibitor Combinations

#### 3.3.1. PD-1/PDL-1-Inhibitors and Anti-CTLA-4 Antibodies

Rationale: Anti-CTLA-4 antibodies, e.g., ipilimumab, promote intratumoral T cell infiltration, but induce the upregulation of the inhibitory immune checkpoints VISTA and PD-L1 within the prostate TME at the same time [134]. Therefore, the simultaneous targeting of both immune checkpoints may help to overcome the adaptive mechanism of immune resistance.

A combination of the anti-CTLA-4 antibody tremelimumab plus PD-L1 inhibitor durvalumab was applied in men with chemotherapy-naïve mCRPC every 4 weeks (up to four doses), followed by durvalumab maintenance every 4 weeks (up to nine doses) in a single-arm pilot study (NCT03204812). Stable disease for >6 months was achieved in 24% of the patients. Median rPFS was 3.7 months (95% CI: 1.9 to 5.7), and median overall survival was 28.1 months (95% CI: 14.5 to 37.3). Of note, post-treatment evaluation of the bone microenvironment revealed transcriptional upregulation in myeloid and neutrophil immune subset signatures and increased the expression of inhibitory immune checkpoints indicating the development of immune resistance [135].

In the CheckMate650 trial, combinational therapy of ipilimumab and nivolumab was evaluated before (cohort A) and after docetaxel treatment (cohort B) in mCRPC (NCT02985957). ORR and disease control rates were 25% and 10%, and 46.9% and 13.3% in the pre- and post-chemotherapy setting, respectively. Two patients in each cohort had complete responses. An OS of 19.0 and 15.2 months was reported for cohort A and B. Initial biomarker analyses suggest improved activity in patients with high TMB (≥74.5 mutations/patient; ORR: 50% vs. 5.3%), evidence of a DDR (ORR: 36.4% vs. 23.1%), and increased PD-L1 expression (≥1%; ORR: 36.4 vs. 12.1%) [136].

A small phase 2 study (n = 15) also evaluated the efficacy of the combination of ipilimumab and nivolumab in patients with evidence of androgen receptor splice variant 7 (AR-V7) (NCT02601014). The rationale for this biomarker-based patient selection was the hypothesis that an increased rate of DNA repair mechanism defects may be present in AR-V7-positive patients. In fact, 40% of the patients carried DDR mutations and outcomes appeared generally better in DDR^+^ compared to DDR^−^ tumors with respect to PSA responses (33% vs. 0%; *p* = 0.14), with ORR (40% vs. 0%; *p* = 0.46) and PSA-PFS (HR 0.19; *p* = 0.11) [137].

In another nonrandomized phase 2 study, the combination of ipilimumab/nivolumab was examined in AR-V7-expressing mCRPC without (Cohort 1) or with (Cohort 2) the anti-androgen enzalutamide. For both groups only modest activity was reported without statistical differences between the cohorts. Lower alkaline phosphatase, lower circulating IL-7 and IL-6 levels, and higher circulating IL-17 levels were associated with improved OS [138]. 

#### 3.3.2. PD-1/PD-L1 Antibodies and Androgen Receptor-Targeting Therapies

Rationale: Androgen receptor inhibitor enzalutamide is assumed to enhance IFNγ signaling and may sensitize tumor cells to immune-mediated cell killing, making it a candidate for combinations with PD-L1/PD-1 inhibitors. In addition, PD-L1 upregulation on dendritic cells in men with mCRPC either progressing on or refractory to enzalutamide has been reported.

In a phase 2 trial, enzalutamide was combined with PD-1 inhibitor pembrolizumab in mCRPC patients progressing on enzalutamide alone (NCT02312557). A PSA decline of ≥50% was reported in 18% of the patients, and 25% of the men with measurable disease at baseline achieved an objective response. Median OS for all patients was 21.9 months (95% CI: 14.7 to 28.4 months) and 41.7 months (95% CI: 22.16 to not reached (NR)) in men responding to the IC/NHA combinational therapy [139]. In addition, cohorts 4 and 5 of the Keynote199 trial (NCT02787005) evaluated pembrolizumab in combination with enzalutamide in chemotherapy-naive mCRPC patients after progression on enzalutamide therapy who had RECIST-measurable (cohort 4) or bone-predominant (cohort 5) disease. In cohort 4, ORR was reported in 12% of the patients. A DCR of 51% was observed for both cohorts. At the time of data cutoff, median OS was not reached in cohort 4 and was 19 months in cohort 5, respectively. Of note, the shorter median OS correlated with prior enzalutamide treatment <6 months [140]. Currently, combinational therapy of pembrolizumab and enzalutamide are compared to enzalutamide alone in phase 3 clinical trials in patients with hormone-sensitive (NCT04191096) or castration-resistant disease (NCT03834493) (Table 2). 

In the IMbassador250 phase 3 clinical trial, a combination of PD-L1 inhibitor atezolizumab and enzalutamide was compared to enzalutamide alone in patients with mCRPC who had progressed on abiraterone and docetaxel or were not candidates for a taxane regimen (NCT03016312). The combination of atezolizumab and enzalutamid did not show an OS improvement (15.2 months (95% CI: 14.0, 17.0) vs. enzalutamide alone (16.6 months (95% CI: 14.7, 18.4), requiring early termination of the study [38,141]. In line with previous findings, low effector T cell and macrophage signatures, as well as reduced MHC class I and immune checkpoint signatures were observed in the majority of patients. In addition, markers of pre-existing immunity, such as PD-L1 IC expression ≥5% (IC2/3), CD8 T cell infiltration, and TMB ≥ 10 mutations per megabase, were rare. However, the presence of PD-L1 IC2/3 expression, high levels of CD8^+^ T cells, and established immune cell signatures were associated with longer PFS in the combination arm. Interestingly, an improved PFS was also found in patients with *PTEN* loss. This is of such interest because loss of PTEN activity is known to be associated with an immunosuppressive milieu (see above). The addition of atezolizumab could potentially reverse this [38].

#### 3.3.3. PD-1/PD-L1 and Chemotherapy

Rationale: Cytotoxic cell death and subsequent antigen release provides immune stimulation, common to conventional and targeted anticancer agents. However, in the past years, there is emerging evidence that the efficacy of chemotherapy does not only involve direct cytostatic/cytotoxic effects, but also relies on the (re)activation of tumor-targeting immune responses [142]. Indeed, a reduction of circulating Treg cells and MDSCs has been described associated with a more immunopermissive TME.

In cohort B of CheckMate9KD, combinational therapy of nivolumab and docetaxel was evaluated in mCRPC patients, who had previously received up to two NHA. An ORR and PSA reduction >50% from baseline was reported for 40% (95% CI, 25.7–55.7) and 46.9% (95% CI, 35.7–58.3) of the patients, respectively. rPFS and OS were 9.0 months (95% CI, 8.0–11.6) and 18.2 months (95% CI, 14.6–20.7), respectively [143,144]. In subpopulations with versus without prior NHA, the ORR was 38.7% versus 42.9% and the PSA reduction >50% was 39.6% vs. 60.7%. In addition, median rPFS was improved from 8.5 to 12.0 months and median OS from 16.2 months versus not reached. Of note, preliminary biomarker analyses revealed no clear association between HRD or TMB with tumor reduction or with decrease in PSA from baseline. In addition, CDK12 mutations did not correlate with response or PSA reduction.

The additional benefit of adding nivolumab or pembrolizumab to chemotherapy with docetaxel are currently being determined in two randomized phase 3 trials (NCT04100018; NCT03834506; Table 2). 

#### 3.3.4. PD-1/PD-L1 and PARP Inhibitors

Rationale: HRR defects have been associated with improved response to immunotherapy in PCa (see above). PARP inhibition may potentiate DNA damage and inefficient repair in tumors, and thus may cause immunologically relevant mutations [145]. 

In a phase 1/2 clinical trial, durvalumab was evaluated in combination with PARP inhibitor (PARPi) olaparib in patients with mCRPC with and without somatic or germline DDR mutations. The median rPFS of patients with alterations in DDR genes was 16.1 months (95% CI: 7.8–18.1 months) with a 12-month PFS probability of 83.3% (95% CI: 27.3– 94.5%) compared with a 12-month probability of 36.4% (95% CI: 11.2–62.7%) for those without mutations; *p* = 0.031). Remarkably, patients’ baseline fraction of MDSCs correlated with response to therapy revealed by a prolonged PFS of those whose percentage of MDSCs among total viable cells was below the median baseline (*p* = 0.041) [146].

Cohort A1 of CheckMate9KD received nivolumab in combination with PARPi rucaparib after 1–2 prior taxane-based chemotherapy regimens and up to 2 NHA for mCRPC (NCT03338790). In HRD^+^ patients, nivolumab and rucaparib achieved a confirmed ORR and a PSA reduction of ≥50% from baseline in 17.2% (95% CI, 5.8–35.8) and 18.2% (95% CI, 8.2–32.7), respectively. In contrast, patients with HRD^−^ tumors did not appear to benefit from either drug [147].

In Cohort A2 of CheckMate9KD, the combination of nivolumab and rucaparib was applied to mCRPC patients without previous taxane treatment and 1–2 prior NHA (NCT03338790). In the pre-chemotherapy setting, confirmed ORR was reported for 25% (95% CI, 8.7–49.1) and a confirmed PSA^−^response for 41.9 (95% CI, 24.5–60.9) of HRD ^+^ patients, respectively. Remarkably, a PSA reduction of >50% from baseline was found in 84.6% of the patients. Again, clinical activity in patients with HRD^−^ tumors was limited [148].

An ongoing randomized phase 3 clinical trial is currently evaluating combinational therapy of pembrolizumab and olaparib compared to the second NHA in HRD-unselected mCRPC patients (NCT03834519; Table 2).

#### 3.3.5. PD-1/PD-L1-Inhibitors and Tyrosinkinase Inhibitors (TKI)

Rationale: Cabozantinib inhibits several tyrosine kinases including MET, VEGF receptors, and TAM family of kinases (TYRO3, MER, and AXL) [149]. Interestingly, it promotes an immune-permissive environment that may enhance response to immune checkpoint inhibitors [150,151].

On the annual meeting of the European Society of Medical Oncology (ESMO) 2021, data of the COSMIC-021 trial cohort 6 were presented, examining the role of cabozantinib and atezolizumab in patients with mCRPC [152]. Pretreatment with at least one NHA was a prerequisite for study inclusion, while chemotherapy was only allowed in the hormone-sensitive setting. At baseline, 77% of the patients had measurable visceral metastases (32%) or extrapelvic lymphadenopathy (EPLN) (60%). ORR per investigator was 23% for all patients and 27% for patients with visceral or EPLN with 2% CR. Remarkably, a disease control rate of 84% and 88% was reported for both groups, respectively. The ongoing phase 3 trial CONTACT-02 evaluates the TKI/CI combination in mCRPC patients with measurable disease who have been pre-treated with one NHA (NCT04446117; Table 2).

In addition, multikinase inhibitor lenvatinib is investigated in combination with pembrolizumab in neuroendocrine PCa (NCT04848337; Table 2).

#### 3.3.6. PD-1/PD-L1 Inhibitors and Radiotherapeutic Approaches

Rationale: Abscopal effects with partial or complete eradication of tumors distant from the local radiation fields have been observed in melanoma and lung cancer patients receiving immunotherapy and local radiotherapy [153,154]. 

In a phase 1b study, PD-L1 inhibitor atezolizumab was combined with radium-223 applying three different treatment schedules (NCT02814669). However, no clear evidence of additional clinical benefit was observed in mCRPC patients with bone and lymph node and/or visceral metastases independent of the regiment used. Of note, the combination was accompanied by increased toxicity compared to either drug alone [155]. Currently, radium-223 is evaluated in combination with nivolumab (NCT04109729), pembrolizumab (NCT03093428), or avelumab and radiation-enhancing medication M3814 (NCT04071236) (Table 2).

Current clinical trials are evaluating the combination of CI with PSMA ligand therapy or metastases-directed therapy (NCT05150236; Table 2). Recently, interim results of the PRINCE trial on the combination of pembrolizumab and radioligand therapy with 177-Lu-PSMA were presented. Here, 37 patients received an average of four doses of 177-Lu-PSMA-617 and eight doses of pembrolizumab. A PSA decline of at least 50% was observed in 73% of patients, and 78% of patients achieved partial remission according to RECIST 1.1. The toxicity profile was similar to that of the single agents. Further results are eagerly awaited.

### 3.4. Bispecific T Cell Engagers

Bispecific T cell engagers (BiTEs) are synthetic proteins designed to activate and target T cells to tumor cells (Figure 2). The structure of BiTEs is based on variable antibody fragments. A BiTE is made up of two different specific binding domains, and each binding domain is formed by two single-chain variable fragments connected by a linker. In general, one of the two domains is specific for CD3, a cell surface marker of T cells that is required for co-stimulation in the T cell receptor complex. The second domain can be specifically adapted to the tumor antigen of interest [156]. Upon binding of the BiTE to the T cell and tumor cell, an immunologic synapse is formed and cytotoxic T cells initiate tumor cell lysis, without the need for further co-stimulation. This is of high value in tumors where MHC class I expression is downregulated, and thus is a promising strategy for PCa [157]. At the immunological synapse perforin and granzymes are released by cytotoxic T cells and eventually cause tumor cell death. As a result of activation, the T cells proliferate, thereby potentiating the antitumor effects [156]. BiTEs are designed to have a higher affinity to tumor-specific targets than to CD3 to reduce binding of T cells in the absence of tumor cells. A reduction of CD3 affinity also decreases cytokine release and, consequently also side effects [158].

Currently, conventional BiTEs must be administered as continuous infusions due to their short half-life. For this reason, extending the half-life is an important scientific challenge to improve clinical applicability. One method to increase half-life time in circulation is the fusion of BiTEs with Fc fragments. For instance, AMG160 is an anti-PSMA half-life extended BiTE that has successfully been tested in animal models and provided first promising clinical results in PCa [159,160]. Thus, at the ESMO annual meeting 2020, preliminary results of an ongoing phase 1 trial were presented (NCT03792841) [160]. At the time of data cutoff, 43 patients had received at least one dose of AMG160 and 44.2% of the patients remained on treatment. However, 95.3% of the patients experienced treatment-related adverse events (TRAE) with three reversible dose-limiting toxicities. Of note, none resulted in treatment discontinuation and no grade 5 events were reported. Confirmed and additional unconfirmed PSA responses (≥30% decrease) were achieved by 27.6% and 11.4% of the patients, respectively. In 23.1% of the men, previously detectable circulating tumor cells disappeared during the course of therapy. Confirmed and unconfirmed responses as well as stable disease according to RECIST1.1 were observed in 13.3%, 6.7%, and 53.3% of the study participants, respectively. A phase 3 trial is currently in preparation. Another approach to overcome the limited half-life time is the use of an injectable polymer depot. Anti-PSMA-BiTEs enclosed in a biopolymer are released as the biopolymer gets slowly degraded. In mouse xenograft models of PCa, the biopolymer showed a low inflammatory potential and the BiTE depots effectively maintained BiTE plasma concentration. Especially in tumors with low PSMA expression, the inhibition of tumor growth was improved with the use of a BiTE depot compared to daily injection of the BiTE alone [159].

An intensive search for alternative target antigens is currently underway, the first of which are now being evaluated in the preclinical setting and early clinical trials. Thus, a BiTE targeting Glypican-1, a heparan sulfate proteoglycan that is overexpressed in PCa with a correlation to the Gleason score, has been designed on the basis of the CD3 binding sequence of blinatumomab in a standard BiTE format. Promising preclinical results have been reported including T cell activation and cytokine release [161]. In addition, a BiTE targeting disintegrin and metalloproteinase 17 (ADAM17), a transmembrane protease, and an anti-ADAM17 BiTEs-mediated specific lysis of ADAM17-expressing cells including PCa cell lines have been analyzed [162]. Prostate stem cell antigen (PSCA), a glycosylphosphatidylinositol (GPI)-anchored cell surface protein, upregulated in different malignancies including mCRPC, is serving as a target for GEM3PSCA, an affinity-tailored T cell adaptor, currently evaluated in PSCA-positive PCa in a phase 1 clinical trial (NCT03927573; Table 3). 

AMG-757 is another half-life extended BiTE targeted against delta-like ligand 3 (DLL3), a notch ligand involved in neuroendocrine differentiation. While AMG-757 has been successfully tested in preclinical models of small cell lung cancer, it might as well be effective against neuroendocrine PCa as DLL3 is also upregulated in these tumors [163,164]. A phase 1 clinical trial is carried out in patients with de novo or treatment emergent neuroendocrine prostate cancer (NCT04702737; Table 3).

A major downside of BiTEs, however, is the activation of immune checkpoint molecules, such as PD-1 or LAG-3, as a consequence of T cell activation. Therefore, combination of BiTEs with immune checkpoint inhibitors might be able to overcome treatment resistance [165]. A recent study in an animal model has revealed that especially immunologically cold tumor with low T cell infiltration may benefit from combination of BiTE therapy and concurrent immune checkpoint inhibition [166].

### 3.5. Chimeric Antigen Receptor T Cells (CAR-T Cells)

Chimeric antigen receptor T cells (CAR-T Cells) are genetically modified T cells that are transfected with a chimeric antigen receptor directed against a tumor antigen (Figure 2). Following in vitro expansion, the CAR-T cells are transfused back into the patient. Optimization of CAR-T constructs mainly focuses on the intracellular signal transduction domains. Common to all is the CD3-zeta signaling domain, while further costimulatory domains have been added in the newer generations to enhance survival and proliferation of CAR-T cells [167,168]. CAR-T cell therapy has already profoundly improved treatment options in adult lymphoblastic leukemia of B cell lineage and of Non-Hodgkin B cell lymphoma as well as multiple myeloma [169,170,171]. In comparison to conventional cytotoxic chemotherapy or immunotherapy with monoclonal antibodies, CAR-T cell therapy has been found capable to induce durable complete responses after a single treatment course. This is based upon the ability of CAR-T cells to expand in vivo and to persist for several years, which leads to continuous therapeutic efficacy and tumor control [168,172]. 

For CAR-T-based therapy of metastatic PCa, several antigens and different CAR-T constructs are currently under clinical investigation (Table 3). PSMA has been identified as an attractive target for CAR-T cell therapy due to its consistent membranous expression and because the majority of mCRPC are positive for PSMA [173]. Of note, PSMA expression has been also described in small intestine, kidney, central nervous system, and salivary glands [174,175]. Therefore, the possible on-target off-tumor toxicity of PSMA-directed CAR-T therapy with regard to these tissues has to be accounted for. Other targets currently under investigation are PSCA and kallikrein 2 (KLK2), both reported with high expression in prostate cancer [176,177,178] (Table 3). Challenges in CAR-T cell therapy of solid tumors and prostate cancer in particular include the previously described immunosuppressive tumor environment as well as reduced homing and decreased persistence of CAR-T cells [179,180]. To counter these barriers, several approaches have been proposed. However, to date only few clinical results are available, mainly reported in press releases or congress abstracts.

In a phase 1 trial, CAR-T cells with specificity to PSMA were co-administered with IL-2 after a non-myeloablative chemotherapy with cyclophosphamide and fludarabine. All toxicities observed were attributed to chemotherapy or IL-2 treatment. Remarkably, despite the early phase 1 study design, two of five patients displayed a PSA response [181]. Another attempt to increase the effectiveness of CAR-T cells is the co-expression of a dominant negative TGF-β receptor in CAR-T cells directed to PSMA. The resulting decrease of the immunosuppressive signaling of TGF beta led to the enhancement of CAR-T proliferation, antitumor activity, and persistence in a pre-clinical model of aggressive PCa [64,182]. Consequently, “augmented” CAR-T cells with a dominant negative TGF-β receptor were evaluated in a phase 1 clinical trial (NCT 03089203). Remarkably, cytokin-release syndrom has been observed as a common toxicity in this trial. This suggests that CAR-T cells were able to withstand the immunosuppressive tumor microenvironment and were stimulated to proliferate by successful binding to the PSMA antigen. Unfortunately, on higher CAR-T doses in this study, excessive toxicity was observed, which led to fatal outcomes in one patient due to neurotoxicity and macrophage activation syndrome. Of note, at this very early stage PSA response was observed in three of six patients [183]. 

Several CAR-T constructs have been developed that harbor safety switches to improve mitigation of toxicities. This includes “on-switches”, which require the presence of a small molecule to enable CAR-T cell activation. For instance, the complete CAR can be formed by two different subunits, one containing only the antigen-binding domain and the other harboring the signal transduction domain within the T cell. Only after infusion of the antigen-binding domain do both subunits dimerize and mediate T cell activation and tumor cell lysis. Withdrawal of the antigen-binding site infusion will disrupt CAR-T cell activation. This concept is currently investigated by the UC02-PSMA trial, which is using a “treatment module” adapter molecule that binds to the target antigen PSMA and the CAR-T cell, which itself is not able to bind to PSMA. The treatment module is given as continuous infusion due to its short half-life. Stopping of the treatment module infusion is expected to rapidly counter CAR-T-mediated toxicities. By using different adaptor molecules with the same CAR epitope, one CAR-T cell population can target multiple tumor-associated antigens [184].

As alternative measure for safety, “off-switches” are used to stop CAR signaling and CAR-T cell proliferation. This includes small molecule inhibitors of T-cell receptor signaling as well as the induction of CAR degradation [184]. Kill switches are permanent off-switches that cause apoptosis of the CAR-T cells upon administration of a small molecule. One example is the transduction of CAR-T cells with a suicide gene, such as the *Herpes simplex* virus thymidine kinase, a mechanism that has so far been used in hematopoietic stem cell transplantations [185]. Additionally, small molecule-mediated dimerization of a transgenic caspase like rimiducid can be applied for CAR-T cell killing [168,186].

Another promising and noteworthy approach of CAR-T therapy is the use of allogeneic rather than the standard autologous T cells for CAR-T manufacturing [187,188]. Besides the logistical advantages of allogeneic CAR-T production, CAR-T cells manufactured from the T cells of healthy donors may provide superior immunological properties and eventually improved efficacy [189]. Although several clinical trials are currently investigating allogeneic CAR-T therapy in hematological und solid malignancies, to the best of our knowledge this is not yet the case for PCa. 

## 4. Future Directions

The start of immunotherapy in the treatment of advanced PCa was bumpy. Many of the high expectations could not be met at first. The growing knowledge about the specific immunosuppressive milieu of PCa and possible counter-regulatory interventions give hope that PCa patients will also be able to benefit from immunotherapy in the future. Various combination therapies to improve the effectiveness of checkpoint inhibitors are currently underway and their results are eagerly awaited.

A perceived setback was the phase 3 results on atezolizumab and enzalutamide showing no OS benefit for the “‘intention to treat” population, and thus requiring early termination of the trial. Nonetheless, important findings on potential biomarkers show that there are PCa patients who will benefit from immunotherapy [38]. In addition, long-term analyses of ipilimumab have recently demonstrated two to three times higher survival rates at 3 years, despite an initially negative result of the trial. Great hope rests on new treatment strategies such as BiTEs or CAR-T cells. However, the recent attempts to counteract immunosuppressive factors by additional genetic modification of CAR-T cells have led to unexpectedly severe toxicities in addition to improved efficacy, which is to be resolved in the future. In conclusion, despite many relevant questions that remain to be addressed, the intensive scientific efforts on different levels give hope that there is a light at the end of the tunnel.

## Figures and Tables

**Table 1:** Active trials examining vaccination strategies in advanced prostate cancer.

Trial Name	Trial Phase	Estimated Enrolment (pts)	Experimental Therapy	Disease Stage	Required Pre-Treatmet	Primary Endpoint	NCT Number
Vaccination (Phase 1/2)							
OVM-200-100	1 FIH	36	OVM-200	mCRPC or locally advanced	Any first-line therapy	Safety, tolerability	NCT05104515
UR1534	1	20	Bcl-xl_42-CAF09b	mHSCP	ADT	Safety	NCT03412786
17-C-0007	1/2	29	PROSTVAC-V/F + Nivo	mCRPC	ADT	Safety	NCT02933255
QuEST1	1/2	113	BN-Brachyury + M7824 vs. BN-Brachyury + M7824 + N-803 vs. BN-Brachyury + M7824 + N-803 + Epacadostat	mCRPC	1 NHA or if MSI high/MMRd: Pembrolizumab or if HRR mutation: Olaparib/Rucaparib	PSA decline of ≥30% (>21 days) and/or OR (RECIST 1.1)	NCT03493945
UW18037	2	60	pTVG-HP + Pembrolizumab vs. pTVG-HP + pTVG-AR + Pembrolizumab	mCRPC	ADT	PFS	NCT04090528
PRO-MERIT	1/2	130	W_pro1 + Cemiplimab	mCRPC	2-3 lines	DLTs TEAEs ORR (Part 2 Arms 1A and 1B)	NCT04382898

FIH, first in human; mCRPC, metastatic castration-resistant prostate cancer; mHNPC, metastatic hormone-naive prostate cancer; ADT, androgen-deprivation therapy; NHA, new hormonal agent; MSI, microsatellite instability; MMRd, mismatch repair deficiency; HRR, homologous recombination repair; DLT, dose-limiting toxicities; TEAEs, treatment-emergent adverse events.

**Table 2:** Active clinical trials on biomarker-selected patients and combinational treatment approaches with checkpoint inhibitors in advanced prostate cancer.

**Trial Name**	**Trial Phase**	**Estimated** **Enrolment** **(pts)**	**Experimental Therapy**	**Disease** **Stage**	**Required Pretreatment**	**Primary Endpoint**	**NCT Number**
CONTACT-02	3	580	Atezolizumab Cabozantinib	mCRPC	1 NHA docetaxel only in HNPC	Duration of PFS by RECIST1.1	NCT04446117
EVOLUTION	2	110	Nivolumab + Ipilimumab + 177 Lu-PSMA	mCRPC	Progression on 1 NHA	PSA-PFS at 1 year	NCT05150236
CheckMate7DX	3	984	Nivolumab + Docetaxel followed by Nivolumab	mCRPC	1-2 NHA	rPFS, OS	NCT04100018
KEYNOTE-991	3	1232	Pembrolizumab + Enzalutamide	mHNPC	Docetaxel in HNPC allowed	rPFS, OS	NCT04191096
KEYNOTE-641	3	1200	Pembrolizumab + Enzalutamide	mCRPC	Chemotherapy-naïve, abiraterone-naïve, or intolerant or progressed on abiraterone	OS, rPFS	NCT03834493
KEYLYNK-010	3	780	Pembrolizumab + Olaparib	mCRPC	1 NHA and Docetaxel	OS, rPFS	NCT03834519
KEYNOTE-921	3	1000	Pembrolizumab + Docetaxel	mCRPC	≤1 NHA or mHSPC or mCRPC	OS, rPFS	NCT03834506
**Trial Name**	**Trial** **Phase**	**Estimated** **Enrolment** **(pts)**	**Experimental Therapy**	**Disease Stage**	**Required Pretreatment**	**Primary Endpoint(s)**	**NCT Number**
AZD4635 in prostate cancer	2	60	Module 1: AZD4635 + durvalumab; Module 2: AZD4635 + oleclumab	mCRPC	Progressed on standard of care	ORR, PSA RR (>50%)	NCT04089553
QUEST (combination 1)	1b/2	136	Cetrelimab + Niraparib	mCRPC	ns	Part 1: incidence of specific toxitities Part 2: ORR	NCT03431350
KRONOS	1b	33	Cetrelimab + Apalutamid	mCRPC	Progression on NHA	Adverse events PSA Response week 12	NCT03551782
ImmunoProst	2	38	Nivolumab	+HRD mCRPC ^1^	docetaxel	PSA RR (>50%)	NCT03040791
PORTER	1	45	A: Nivolumab + NKTR-214 B: Nivolumab + SBRT + CDX-301 + Poly-ICLC C: Nivolumab + CDX-301 + INO-5151	mCRPC	Prior NHA (e.g., abiraterone, enzalutamide, apalautamide)	Incidence and severity of adverse events	NCT03835533
201808043 CA209-9MW	1	20	Nivolumab/Ipilimumab/ PROSTVAC/Neoantigen DNA vaccine	mHNPC	Chemohormonal therapy	Safety and Tolerability	NCT03532217
CA209-935	2	175	Nivolumab + Ipilimumab (4 times) followed by Nivolumab maintenance	mCRPC with immunogenic signature ^2^	1 line of therapy	Composite response rate ^3^	NCT03061539
IMPACT CA209-8JJ (cohort A)	2	40	Nivolumab + Ipilimumab (4 times) followed by Nivolumab maintenance	mCRPC with CDK12 mutations	ns	PSA RR (>50%)	NCT03570619
INSPIRE CA184-585	2	75	Nivolumab + Ipilimumab (4 times) followed by Nivolumab monotherapy	mCRPC with immunogenic phenotype ^4^	ns	DCR ^5^	NCT04717154
Rad2Nivo CA209-7G6	1b/2	36	Nivolumab + Radium223	Symptomatic mCRPC without visceral Mets	ns	Safety ctDNA reduction after 6 weeks	NCT04109729
PLANE-PC	2	50	Pembrolizumab + Lenvatinib	Neuroendocrine PCa	ns	rPFS	NCT04848337
Keynote 365	1b/2	1000	Cohort A AC: Pembrolizumab + Olaparib Cohort B AC: Pembrolizumab + Docetaxel + Prednisone Cohort C AC: Pembrolizumab + Enzalutamide Cohort D AC: Pembrolizumab + Abiraterone + Prednisone Cohort E AC: Pembrolizumab + Lenvatinib Cohort F t-NE: Pembrolizumab + Lenvatinib Cohort G (AC) Pembrolizumab/Vibostolimab coformulation Cohort H t-NE: Pembrolizumab/Vibostolimab coformulation Cohort I t-NE: Pembrolizumab + Carboplatin + Etoposide	For Cohorts A, B, C, D, E, and G: histologically or cytologically confirmed adenocarcinoma of the prostate without small cell histology Cohorts F, H, and I: neuroendocrine PCa defined by ≥1% neuroendocrine cells in a recent biopsy specimen	Cohort E: Docetaxel + up to 2 NHA Cohort F, G, H, I: Docetaxel + 1 other chemotherapy allowed + up to 2 NHA	50% PSA RR ORR Number of participants with AEs Number of participants discontinuing study medication due to AEs	NCT02861573

^1^ BRCA1, BRCA2, ATM, PTEN, CHEK2, RAD51C, RAD51D, PALB2, MLH1, MSH2, MSH6, and PMS2; ^2^ Immunogenic signature: mismatch repair deficiency by IHC, defective DNA repair detected by a targeted sequencing panel, and high inflammatory infiltrate defined on multiplexed IHC criteria; ^3^ Composite response rate: radiological response (RECIST 1.1), PSA response ≥50% confirmed by a second PSA test at least 4 weeks later (PCWG3 2016), and conversion of CTC count from ≥5 cells/7.5 mL at baseline to <5 cells/7.5 mL confirmed by a second CTC test at least 4 weeks later (PCWG3 2016). ^4^ Immunogenic phenotype with of one of the next criteria: 1, mismatch repair deficiency and/or a high mutational burden of >7 mutations per Mb (cluster A); 2, BRCA2 inactivation or BRCAness signature (cluster B); 3, a tandem duplication signature and/or CDK12 biallelic inactivation (cluster C). ^5^ Disease control rate (DCR) of >6 months; this includes a change from baseline in tumor volume as measured by SD, PR, or CR by best ORR in evaluable participants, all lasting longer than 6 months; Abbreviations: mCRPC: metastatic castration resistant prostate cancer, mHNPC: metastatic hormone naïve prostate cancer; ns not specified AC: adenocarcinoma; t-NE: transdifferentiated neuroendocrine carcinoma of the prostate.

**Table 3:** Active trials with bispecific T cell engagers (BiTEs) and CAR-T cells in advanced PCa.

Short trial Title	Trial Phase	Estimated Enrolment (pts)	Experimental Therapy	Disease Stage	Required Pretreatment	Primary Endpoint	NCT Number
Safety, Tolerability, Pharmacokinetics, and Efficacy of Acapatamab in Subjects With mCRPC	1	288	Acapatamab, acapatamab + Pembrolizumab, acapatamab + Etanercept Prophylaxis, acapatamab + Cytochrome P450 Cocktail	mCRPC	ADT, taxane	Safety and tolerability	NCT03792841
A Study of Tarlatamab (AMG 757) in Participants with Neuroendocrine Prostate Cancer	1b	60	Tarlatamab (AMG 757)	Neuroendocrine prostate cancer	1 line of prior systemic treatment	Safety and tolerability	NCT04702737
Study of AMG 509 in Subjects with Metastatic Castration-Resistant Prostate Cancer	1	110	AMG 509	mCRPC	Prior NHA, taxane	Safety and tolerability	NCT04221542
Safety and Efficacy of Therapies for Metastatic Castration-Resistant Prostate Cancer (mCRPC)	1/2	159	Acapatamab + Enzalutamide, Acapatamab + Abiraterone, Acapatamab + AMG 404	mCRPC		Safety and tolerability	NCT04631601
Study with Bispecific Antibody Engaging T cells, in Patients with Progressive Cancer Diseases With Positive PSCA Marker	1	24	GEM3PSCA	PSCA expressing cancer including prostate carcinoma	Progressive Disease After Standard Systemic Therapy	MTD Incidence and intensity of AEs DLT	NCT03927573
CART-PSMA-TGFβRDN Cells for Castrate-Resistant Prostate Cancer	1	18	CART-PSMA-TGFβRDN	mCRPC	At least 1 NHA	Safety and tolerability	NCT03089203
P-PSMA-101 CAR-T Cells in the Treatment of Subjects With mCRPC and Advanced Salivary Gland Cancers	1	60	P-PSMA-101 Rimiducid (safety switch activator) may be administered as indicated	mCRPC		Safety, DLT, efficacy RECIST 1.1 and PCWG3	NCT04249947
PSCA-CAR T Cells in Treating Patients with PSCA + mCRPC	1	33	Autologous Anti-PSCA-CAR-4-1BB/TCRzeta-CD19t-expressing T-lymphocytes	mCRPC	At least 1 NHA	Safety and tolerability Define recommended phase 2 dose	NCT03873805
Safety and Activity Study of PSCA-Targeted CAR-T Cells (BPX-601) in Subjects with Selected Advanced Solid Tumors	1/2	151	BPX-601: Autologous T cells genetically modified with retrovirus vector containing PSCA-specific CAR and an inducible MyD88/Cluster designation (CD)40 (iMC) co-stimulatory domain Rimiducid: Dimerizer infusion to activate the iMC of the BPX-601 cells for improved proliferation and persistence	mCRPC among others		MTD and/or recommended extension dose of BPX-601 measured by DLT	NCT02744287
A Study of JNJ-75229414 for Metastatic Castration-Resistant Prostate Cancer Participant	1	60	KLK2 CAR-T Cells (JNJ-75229414)	mCRPC	At least 1 NHA or one prior chemotherapy	Number and severity of AE, DLT	NCT05022849
Dose-Escalating Trial with UniCAR02-T Cells and PSMA Target Module (TMpPSMA) in Patients with Progressive Disease After Standard Systemic Therapy in Cancers With Positive PSMA Marker	1	35	UniCAR02-T Cells and PSMA Target Module (TMpPSMA)	mCRPC	Systemic standard therapies	Safety and tolerability, MTD, DLT	NCT04633148

Abbreviations: MTD: maximum tolerated dose, AE: adverse event; DLT: dose-limiting toxicity.

## Abbreviations

CTLA-4	cytotoxic T lymphocyte antigen 4
PD-1	programmed death-1
PD-L1	PD-1 ligand
PCa	prostate cancer
TME	tumor microenvironment
BiTE	bispecific T cell engager
CAR-T cells	chimeric antigen receptor T cells
TMИ	tumor mutational burden
MHC	major histocompatibility complex
CTL	cytotoxic T lymphocytes
TILs	tumor-infiltrating lymphocytes
DDR	DNA damage repair
MMR	mismatch repair
MSI	microsatellite instability
HRR	homologous recombination repair
Treg	regulatory T cells
AVPC	aggressive variant of prostate cancer
MDSCs	myeloid-derived suppressor cells
AK	androgen receptor
ADT	androgen-deprivation therapy
TAM	tumor-associated macrophages
TNF-α	tumor necrosis factor-α
MSCs	mesenchymal stromal cells
CAFs	cancer-associated fibroblasts
EMT	epithelial–mesenchymal transition
PAP	prostatic acid phosphatase
ATP	adenosine triphosphate
DAMP	danger-associated molecular pattern
TAA	tumor-associated antigens
PSA	prostate-specific antigen
PSMA	prostate-specific membrane antigen
PSCA	prostate stem cell antigen
APC	antigen-presenting cell
DC	dendritic cell
OS	overall survival
mCRPC	metastatic castration-resistant prostate cancer
PBMCs	peripheral blood mononuclear cells
mHNPC	metastatic hormone-naïve prostate cancer
PPV	personalized peptide vaccination
PFS	progression-free survival
CI	immune checkpoint inhibitor
ORR	overall response rate
SD	stable disease
DCR	disease control rate
PR	partial response
HRD	homologous recombination deficiency
TRAE	Treatment-related adverse events
